# Simultaneous Qualitation and Quantitation of Chlorogenic Acids in Kuding Tea Using Ultra-High-Performance Liquid Chromatography–Diode Array Detection Coupled with Linear Ion Trap–Orbitrap Mass Spectrometer

**DOI:** 10.3390/molecules21121728

**Published:** 2016-12-16

**Authors:** Yanyun Che, Zhibin Wang, Zhiyun Zhu, Yangyang Ma, Yaqiong Zhang, Wen Gu, Jiayu Zhang, Gaoxiong Rao

**Affiliations:** 1 Engineering Laboratory for National Healthcare Theories and Products of Yunnan Province, College of Pharmaceutical Science, Yunnan University of Traditional Chinese Medicine, Kunming 650500, Yunnan, China; checpu@163.com (Y.C.); zzy299106@163.com (Z.Z.); mdmy0622@163.com (Y.M.); zzyyqq2003@163.com (Y.Z.); kaoyanak@163.com (W.G.); 2 Beijing Research Institution of Chinese Medicine, Beijing University of Chinese Medicine, Beijing 100029, China; wangzhibin4804@126.com

**Keywords:** chlorogenic acids (CGAs), UHPLC-LTQ-Orbitrap MS, Kuding tea (*Ilex Kudingcha*)

## Abstract

Kuding tea, the leaves of *Ilex Kudingcha* C.J. Tseng, has been applied for treating obesity, hypertension, cardiovascular disease, hyperlipidemia, and so on. The chlorogenic acids (CGAs) in Kuding tea have shown excellent antioxidative, antiobesity, anti-atherosclerotic and anticancer activities. Nevertheless, the chemical profiles of CGAs in Kuding tea have not been comprehensively studied yet, which hinders further quality control. In the present study, a sensitive ultra-high-performance liquid chromatography-diode array detection coupled with a linear ion trap-Orbitrap (UHPLC-DAD-LTQ-Orbitrap) method was established to screen and identify CGAs in Kuding tea. Six CGA standards were first analyzed in negative ion mode with a CID-MS/MS experiment and then the diagnostic product ions (DPIs) were summarized. According to the retention behavior in the RP-ODS column, accurate mass measurement, DPIs and relevant bibliography data, a total of 68 CGA candidates attributed to 12 categories were unambiguously or preliminarily screened and characterized within 18 min of chromatographic time. This was the first systematic report on the distribution of CGAs in Kuding tea. Meanwhile, the contents of 6 major CGAs in Kuding tea were also determined by the UHPLC-DAD method. All the results indicated that the established analytical method could be employed as an effective technique for the comprehensive and systematic characterization of CGAs and quality control of the botanic extracts or Chinese medicinal formulas that contain various CGAs.

## 1. Introduction

Kuding tea, the leaves of *Ilex Kudingcha* C.J. Tseng (Aquifoliaceae), has been used in China for more than 2000 years as tea products. It is traditionally applied for treating obesity, hypertension, cardiovascular disease, hyperlipidemia and various other diseases [1]. Meanwhile, the different extracts and active components from Kuding tea, including triterpenes, triterpenoid saponins and chlorogenic acids (CGAs), have been reported to possess significant antioxidative [2,3], antiobesity [4], antidiabetic [5,6], anti-inflammatory [7], anti-atherosclerotic [8] and anticancer activities [9] in vitro or in vivo. CGAs are a large family of esters formed between quinic acid and one to four residues of certain cinnamic acids, most commonly caffeic, *p*-coumaric and ferulic [10,11]. The distinctive characteristic of CGAs is that they usually have many isomers owing to the different substituted positions of cinnamic acids on quinic acid. In the previous work, the isolation and structural identification of only 13 phenolic acids from Kuding tea have been reported [3]. A high-performance liquid chromatography (HPLC) method with DAD has also been developed for the simultaneous determination of 6 CGA derivatives [12]. However, the determination method by HPLC usually took too much time (>40 min), and was not sensitive enough for trace component analysis in the complicated extracts. 

Recently, a hybrid linear ion trap-Orbitrap (LTQ-Orbitrap) analytical platform has been applied to the analysis of small molecules in various traditional Chinese medicine (TCM) and biological samples [13,14,15,16,17]. It consists of a 2D ion trap coupled with an Orbitrap, and allows two different scan types to be acquired simultaneously. The Orbitrap mass spectrometer, otherwise defined as an electrostatic Fourier Transform mass spectrometer, provides a higher mass resolution and mass accuracy than any other electrostatic mass spectrometers [18]. The ion trap can provide multi-stage MS^n^ mass spectra using data-dependent analysis while an Orbitrap scan can achieve mass accuracies of <5 ppm in an external calibration mode. A full scan mass spectrum acquired with a mass resolution of 30,000 for Orbitrap needs 0.4 s, and provides 25 data points across a peak of width at baseline of 10 s. This advantage facilitates the identification of known and novel constituents in TCMs.

In the present study, a rapid and specific ultra-high-performance liquid chromatography-diode array detection coupled with LTQ-Orbitrap mass spectrometer (UHPLC-DAD-LTQ-Orbitrap MS) method for identification of the characteristic CGAs in Kuding tea was developed. In addition, an accurate and valid method has been established by UHPLC-DAD to simultaneously determine six major CGAs in Kuding tea.

## 2. Results and Discussion

### 2.1. Optimum Conditions for UHPLC-DAD-LTQ-Orbitrap MS Analysis

**Figure 1:**
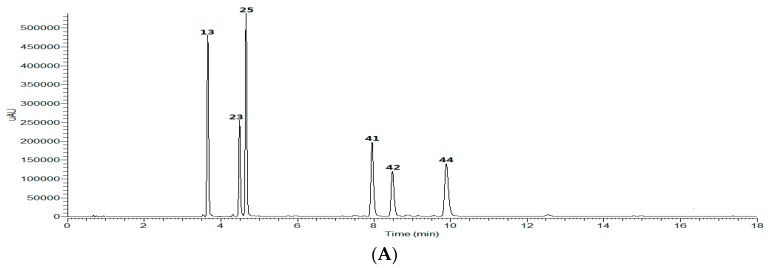

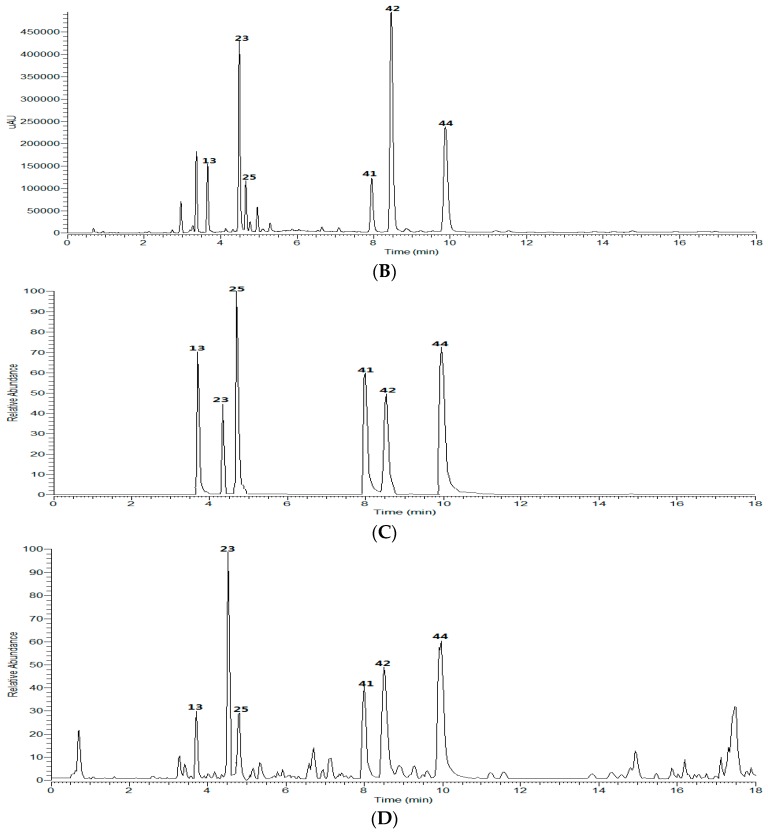
UHPLC-DAD-LTQ-Orbitrap analysis of CGAs in Kuding tea (2 μL): UHPLC-DAD chromatogram of reference standards (**A**) and the extract (**B**) at 327 nm; the total ion chromatogram (TIC) of reference standards (**C**) and the extract (**D**) in negative mode.

To obtain a satisfactory analytical method, chromatographic conditions including mobile phase, flow rate, formic acid addition and column type were all optimized after several trials. DAD detection was employed to monitor the analytes with wavelengths from 200 nm to 400 nm. It was found that detection at 327 nm could provide an optimal signal-to-noise ratio for simultaneously quanlitive and quantitative analysis of CGAs in Kuding tea (Figure 1A,B). Meanwhile, all the factors related to MS performance, including ionization mode, sheath gas flow rate, aux gas flow rate, spray voltage of the ion source and collision energy have been investigated. The results demonstrated that ESI in negative ion mode was more sensitive than in positive ion mode, which was in accordance with the fact that the substances under investigation are phenolic acids with some hydroxyl groups. The major CGAs in Kuding tea were well detected, and exhibited [M − H]^−^ ions and product ions with abundant structural information (Figure 1C,D).

ESI-MS conditions were optimized on an LTQ-Orbitrap MS instrument using the standard solution of 5-Caffeoylquinic acid (5-CQA, 10 μg/mL). To achieve the optimized collision energy that generated adequate fragment information for the structural elucidation and characterization, a series of ESI-MS/MS experiments were carried out at different collision energy (CE, 10%–100%). By gradually increasing the CE, the intensity of product ions was first increased to maximum and then gradually decreased. Even though the optimum CE might vary for different CGAs, the result demonstrated that 35% CE was sufficient to yield abundant fragment ions for the structural elucidation.

### 2.2. Determination of DPIs for CGAs Identification

In the previous reports, diagnostic product ions (DPIs) of CGAs have been summarized based on the high-resolution and low-resolution MS data acquired [15,19]. For example, DPIs of CQAs were determined to be fragment ions at *m*/*z* 191 ([quinic acid − H]^−^), 179 ([caffeic acid − H]^−^) and 173 ([quinic acid − H − H_2_O]^−^). Meanwhile, fragment ions at *m*/*z* 677 corresponding to [Tricaffeoylquinic acids (TriCQA) − H]^−^, *m*/*z* 515 corresponding to [Dicaffeoylquinic acids (DiCQA) − H]^−^ and *m*/*z* 353 corresponding to [CQA − H]^−^ were determined as the additional DPIs. Considering that CGAs are a series of esters formed by quinic acid and certain cinnamic acid, the fragmentation patterns should be similar with those of CQAs. Thus, the cinnamic acid moiety, quinic acid moiety, H_2_O, and CO should be common chemical groups to be easily eliminated from [M − H]^−^ ions of CGAs to afford their respective DPIs.

### 2.3. Characterization of Isomeric Monoacyl CGAs and Their Glycosides

Peaks 1–3 eluted with short retention time and high hydrophilicity yielded their respective [M − H]^−^ ions at *m*/*z* 353.1078 (C_13_H_21_O_11_, <5 ppm) and DPIs such as *m*/*z* 191 ([M − H − Glc]^−^) and *m*/*z* 173 ([M − H – Glc − H_2_O]^−^). Therefore, they were tentatively attributed to Quinic acid (QA) glycosides. 

Peaks 11, 13, 23 and 25 all produced the same [M − H]^−^ ions at *m*/*z* 353.0868 within 5 ppm error, and thus they were tentatively identified as CQAs. For their structural identification, the linkage position of caffeoyl groups on quinic acid could be determined according to the relative intensities of ESI-MS^2^ base peak ion and dominant product ions [20]. When the caffeoyl group was linked to quinic acid at 3-OH or 5-OH, *m*/*z* 191 was the base peak ion, and *m*/*z* 179 was much more significant for 3-CQA. While *m*/*z* 173 was the prominent peak, the caffeoyl group was linked at 4-OH. Therefore, peaks 13 and 25 were respectively identified as 3-CQA and 4-CQA, which were further confirmed by the reference standards. As for peaks 11 and 23, it was nearly impossible to reliably distinguish them from each other only based on their fragmentation patterns. However, the available reference standard enabled both of them to be characterized as 1-CQA and 5-CQA, respectively.

Peaks 20, 27 and 30 all generated the same [M − H]^−^ ions at *m*/*z* 337.0928 within 5 ppm error. In their ESI-MS/MS spectra, they generated their respective ESI-MS^2^ base peak ion at *m*/*z* 163 ([coumaric acid − H]^−^), 191 ([quinic acid − H]^−^ ) and *m*/*z* 173 ([quinic acid – H − H_2_O]^−^ ). According to the MS/MS fragmentation patterns and published DPIs [21], they were eventually characterized to be 3-*p*-Coumaroylquinic acid (3-*p*CoQA), 5-*p*CoQA and 4-*p*CoQA, respectively. 

Peaks 26, 28 and 33 attributed to Feruloylquinic acids (FQA) gave the same [M − H]^−^ ions at *m*/*z* 367.1024 (C_17_H_19_O_9_, <5 ppm). In the previously report [22], 5-FQA produced MS^2^ base peak ion at *m*/*z* 191 accompanied by a weak ion at *m*/*z* 173, while 4-FQA and 3-FQA respectively generated MS^2^ base peak ion at *m*/*z* 173 and *m*/*z* 193. Therefore, according to the DPIs and bibliography data, they were tentatively assigned as 3-FQA, 5-FQA and 4-FQA, respectively.

The sugar residue(s) in CQA glycosides could make them much more hydrophilic than the second/third caffeic acid residue. Coupled with the accurate mass [M − H]^−^ ions at *m*/*z* 515.1395 within 5 mass error and the important DPI at *m*/*z* 353 ([M − H − glucose]^−^), peaks 4, 6, 8, 10, 14, 15, 17, 18 and 21 could be attributed to CQA glycosides. The deficiency of further fragmentation of [M − H − glucose]^−^ made the link position of caffeic acid moiety on quinic acid determination impossible, which was most likely caused by their low contents in Kuding tea extract. Likewise, the [M − H]^−^ ions of peaks 5, 7, 9, 12 and 16 all yielded dominant fragment ions at *m*/*z* 515, *m*/*z* 353, *m*/*z* 191, *m*/*z* 179 and *m*/*z* 173. Furthermore, their short retention time on RP-ODS chromatographic column suggested these five CGA candidates could be interpreted to be CQA diglycosides.

### 2.4. Characterization of Isomeric Diacyl CGAs and Their Glycosides

There were no less than 7 chromatographic peaks which afforded [M − H]^−^ ions at *m*/*z* 515.1184 (C_25_H_23_O_12_, <5 ppm). Therefore, they were preliminarily interpreted as DiCQA. In the ESI-MS/MS experiment, their [M − H]^−^ ions all produced the significant DPI at *m*/*z* 353 ([CQA − H]^−^), which further confirmed the deduction above. Their ESI-MS^3^ spectra were significantly different, which could provide a relatively accurate structural characterization based on the DPIs. Peaks 40, 42 and 43 all produced their ESI-MS^3^ base peak ion at *m*/*z* 191 and secondary peak at *m*/*z* 179 (>40%), which indicated that they could be characterized as 3-substituted quinic acids. According to the eluted orders on the RP-ODS chromatographic column and the comparison with reference substance, 42 was unambiguously identified as 3,5-DiCQA, while the other two peaks were tentatively assigned to be 1,3-DiCQA and *cis*-1,3-DiCQA, respectively. Both peaks 41 and 44 produced the ESI-MS^3^ base peak at *m*/*z* 173, which suggested they might be identified as 4-substituted quinic acids. By comparing with the retention time and fragmentation pathway of the corresponding reference standards, they were unambiguously identified as 3,4-DiCQA and 4,5-DiCQA, respectively. Peak 48 was characterized as 1,5-DiCQA according to the presence of base peak at *m*/*z* 191 and minor peak at *m*/*z* 179. Meanwhile, peak 64 was tentatively interpreted to be 1,4-DiCQA, which was consistent with the secondary peak at *m*/*z* 173 in its ESI-MS^3^ spectrum.

Peaks 45–46, 50–51, 53, 59 and 61 all afforded the same [M − H]^−^ ions at *m*/*z* 499.1235 (C_25_H_23_O_11_, <5 ppm). The DPIs such as *m*/*z* 353 ([CQA − H]^−^) and/or *m*/*z* 337 ([*p*CoQA − H]^−^) coupled with the [M − H]^−^ ions obtained indicated that these CGA candidates could be deduced to be *p*-Coumaroylcaffeoylquinic acids (*p*CoCQAs). Likewise, nine chromatographic peaks including 49, 52, 54, 56, 58, 63 and 65–67 all generated the same [M − H]^−^ ions at *m*/*z* 529.1341 (C_26_H_25_O_12_, <5 ppm). In the ESI-MS/MS experiment, *m*/*z* 529 underwent further fragmentation and yielded several DPIs such as ([CQA − H]^−^) and/or *m*/*z* 367 ([FQA − H]^−^), suggesting they could be interpreted as Caffeoylferuloylquinic acids (CFQAs). In theory, for their structural characterization, the linkage position of caffeoyl, coumaroyl or feruloyl groups on quinic acid could be determined according to the DPIs. However, owing to the low contents of *p*CoCQAs and CFQAs existing in Kuding tea, the further fragmentations of *m*/*z* 367, *m*/*z* 353 or *m*/*z* 337 did not occur in the ESI-MS/MS experiment. Therefore, these 16 CGA candidates were only tentatively characterized as *p*CoCQAs and CFQA, respectively.

Neutral elimination of a glucose unit (162 Da) in the pyran ring is a typical fragmentation pathway for DiCQA glycosides characterization. In combination with the observation of neutral losses such as caffeoyl moiety, quinic acid residue, H_2_O, and CO, nine chromatographic peaks (29, 31–32 and 34–39) were rapidly identified as DiCQA glycosides. In the same way, peaks 19, 22 and 24 afforded the same [M − H]^−^ ions at *m*/*z* 691.1869 (C_32_H_35_O_17_, <5 ppm). The fragment ions such as *m*/*z* 529 ([CFQA − H − Glu]^−^), *m*/*z* 353 ([CQA − H]^−^) and/or *m*/*z* 367 ([FQA − H]^−^) suggested that these three CGA candidates could be deduced to be CFQA glycosides.

### 2.5. Characterization of Isomeric Triacyl CGAs

On the basis of the accurate mass of [M − H]^−^ ions *m*/*z* 677.1501 (C_34_H_29_O_15_, <5 ppm), 7 chromatographic peaks including 47, 55, 57, 60, 62 and 68–69 were tentatively assigned to be TriCQAs (Tricaffeoylquinic acids). Since TriCQAs could be biosynthesized by the polymerization of three caffeic acids and one quinic acid, they often yielded the MS/MS fragment ions including *m*/*z* 515, *m*/*z* 353, *m*/*z* 191, *m*/*z* 179 and *m*/*z* 173, etc. The observation of those DPIs in their ESI-MS/MS spectra further confirmed our deductions.

### 2.6. The Analytical Method Validation and Application

Validation of the established chromatographic method was assessed by several analytical parameters. For determination of six major CGAs in Kuding tea, a calibration curve for each marker was constructed and tested thrice for linearity. As shown in Table 1 good linearity and high sensitivity under the optimal chromatographic conditions were obtained with correlation coefficients no less than 0.9999 and relative low limits of detection (LOD, 0.132–1.108 ng) and limits of quantification (LOQ, 0.431–3.232 ng).

As demonstrated in Table 2, the results of precision and accuracy showed good reproducibility for quantification of 6 CQAs with intra- and inter-day variation less than 1.74% and 1.58%, respectively. The RSDs (relative standard deviations) of the repeatability experiments were less than 1.27% for all analytes. The overall recoveries of the six investigated compounds ranged from 96.40% to 101.61% with RSDs from 1.63% to 2.85%. 

Six major CGAs in 8 batches of Kuding tea (S1–S8) collected from different geographical locations were simultaneously determined by the proposed UHPLC-DAD method. Each sample was analyzed in triplicate, and the results are summarized in Table 3.

### 2.7. Discussion

**Figure 2:**
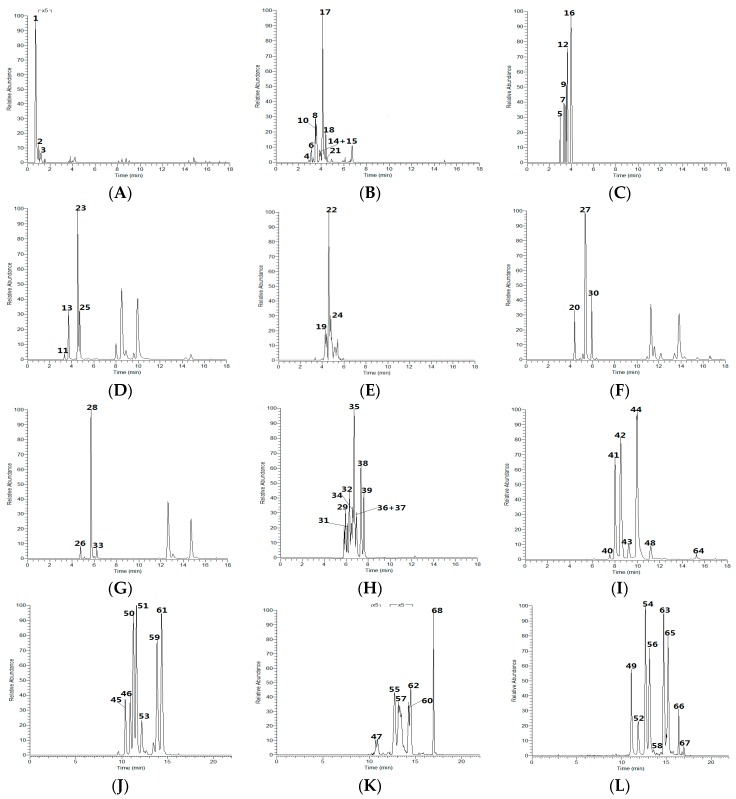
The distributions of 68 CGAs attributed to 12 categories in Kuding tea (**S1**). (**A**) QA-Glc; (**B**) CQA-Glc; (**C**) CQA-DiGlc; (**D**) CQA; (**E**) CFQA-Glc; (**F**) *p*-CoQA; (**G**) FQA; (**H**) DiCQA-Glc; (**I**) DiCQA; (**J**) *p*-CoCQA; (**K**) TriCQA; (**L**) CFQA.

**Figure 3:**
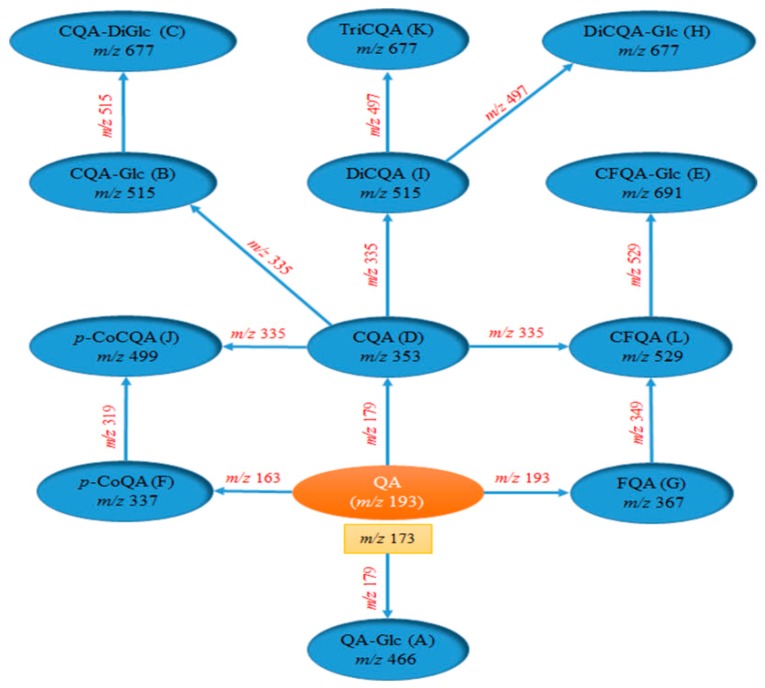
The DPI network of 12 categories of CGAs identified in the present study.

As a result, a total of 68 CGA candidates attributed to 12 categories were identified, including 3 *p*CoQAs, 4 CQAs, 3 FQAs, 3 QA glycosides, 7 *p*CoCQAs, 7 DiCQAs, 9 CQA glycosides, 9 CFQAs, 6 TriCQAs, 9 DiCQA glycosides, 5 CQA diglycosides, and 3 CFQA glycosides. Among them, 6 CQAs were unambiguously identified by comparison with reference standards (Table 4, Figure 2). Owing to the low contents in the Kuding tea extract, many significant ESI-MS/MS fragment ions of CGAs could not be obtained in the experiment, which make it difficult discriminate them from one another. Furthermore, the DPIs network of 12 categories of CGAs was also illustrated in Figure 3 based on the screening and identification results in the present study, which could be worthwhile for systematic identification in the other botanic plants and TCMs.

Meanwhile, six major CGAs tested were detected in all samples with a significant variation in the contents. For instance, 3,5-DiCQA, the highest constituents among the investigated compounds, varied from 23.94 mg/g (S7) to 52.18 mg/g (S6), which indicated that there were great variations between the samples from different sources, even for the samples from the same province. A number of factors may contribute to the variation of the CGA contents among samples, such as their origins, geographical climate and environment, storage conditions, harvest time, cultivated years, drying process, and so on.

## 3. Materials and Methods

### 3.1. Materials and Chemicals

**Figure 4:**
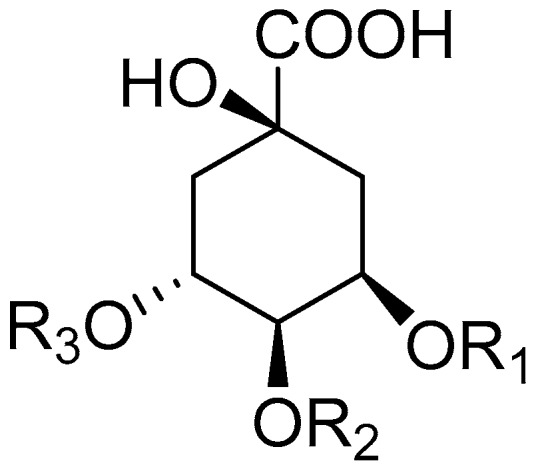
Structures of 6 CGA reference standards identified from Kuding tea.

Six CGA reference standards including 3-O-caffeoylquinic acid (3-CQA), 4-O-caffeoylquinic acid (4-CQA), 5-O-caffeoylquinic acid (5-CQA), 3,4-diCQA, 3,5-diCQA and 4,5-diCQA were purchased from Must Bio-technology Co. Ltd. (Chengdu, China). Their structures (shown in Figure 4) were fully elucidated by the comparison of their spectra data (ESI-MS and ^1^H, ^13^C-NMR) with those published literature values [23]. Their purities were determined to be no less than 98% by HPLC-UV at 327 nm.

Kuding tea made from *I. Kudingcha* was obtained from Guangxi Daxin Bio-Technology Co., Ltd., Guangxi, China (S1–S4, voucher no. 20150101–20150104), Hainan Yexian Bio-Science Technology Co., Ltd., Hainan, China (S5–S7, voucher no. 20160101–20160103), and Cao-bang province, Northern Vietnam (S8, voucher no. 20141001). All samples were authenticated as the leaves of *Ilex Kudingcha* by Professor Deqiang Feng. The voucher specimens of materials were deposited at the department of medicinal chemistry, Yunnan University of Traditional Chinese Medicine.

HPLC-grade acetonitrile and methanol were purchased from Fisher Scientific (Fair Lawn, NJ, USA). Formic acid was purchased from Sigma Aldrich (St. Louis, MO, USA). Deionized water used throughout the experiment was purified by a Milli-Q Gradient A 10 System (Millipore, Billerica, MA, USA). The 0.22 µm membranes were purchased from Xinjinghua Co. Ltd. (Shanghai, China). 

### 3.2. Standard Solutions and Sample Preparation

Each reference compound was accurately weighed, dissolved in methanol, and serially diluted to produce the calibration curves, check linearity, and determine LOD as well as LOQ. All the standard solutions were stored in the refrigerator at 4 °C prior to analysis.

Dried powders of Kuding tea were weighed accurately (0.20 g) and placed into a 50 mL flask containing 25 mL of methanol/water (70:30, *v*/*v*). Then the mixture was extracted in ultrasonic bath (Eima Ultrasonics Corp., Darmstadt, Germany) at room temperature for 30 min. The resulting mixture was filtered through a 0.22 µm membrane, and 2 µL of the filtrate was injected into LC-MS system for analysis.

### 3.3. UHPLC-DAD-LTQ-Orbitrap MS^n^ Analysis

The UHPLC-DAD quantitative analysis was carried out on UltiMate™ 3000 UHPLC system (Thermo Scientific, Bremen, Germany), equipped with a binary pump, an auto sampler, a photo-diode array detector and a column temperature controller. The analytical column was an Acquity BEH C_18_ (1.7 μm, 2.1 × 100 mm i.d.) with the oven temperature maintained at 35 °C. A mobile phase composed of eluent A (0.1% formic acid in water, *v*/*v*) and B (0.1% formic acid in acetonitrile, *v*/*v*) with a linear gradient set as follows: 0 min, 3% B; 4 min, 19% B; 10 min, 20% B; 13 min, 21% B; 17 min, 40% B; 20 min, 90% B. The flow rate was at 0.40 mL/min and peaks were detected at 327 nm. 

HRMS and MS/MS spectral analysis were performed on LTQ-Orbitrap mass spectrometer (Thermo Scientific, Bremen, Germany), which was connected to UHPLC instrument via an ESI interface. Samples were analyzed in negative ion mode with the ion-source parameters set as follows: sheath gas at 25 arb, auxiliary gas at 3 arb, spray voltage at 4 kV, capillary temperature at 320 °C, tube lens at 120 V and capillary voltage at 30 V. Accurate mass analysis was calibrated according to the manufacturer’s guidelines. The MS data were collected at *m*/*z* 100–800. A high-resolution scan was conducted using the Orbitrap to acquire ESI-MS data at a resolution at 30,000 FWHM, and the LTQ dynode was used for scanning ESI-MS^n^ spectra. The dynamic exclusion function was used to reduce the repeat scans, and the repeat count was 2. The exclusion duration was 20 s, and the exclusion mass width was 2 *m*/*z*. The collision energy for collision-induced dissociation (CID) was adjusted to 35% of maximum. 

### 3.4. Analytical Methods Validation

The mixed standard solution containing 97.8 μg/mL of 3-CQA, 142.6 μg/mL of 5-CQA, 162.3 μg/mL of 4-CQA, 141.2 μg/mL of 3,4-diCQA, 106.0 μg/mL of 3,5-diCQA, and 171.4 μg/mL of 4,5-diCQA were prepared in a volumetric flask. These solutions were stored in dark glass bottles at 4 °C. The working standard solutions were freshly prepared by diluting suitable amounts of the above solutions with methanol before injection.

Calibration curves were plotted by the peak area versus at least six appropriate concentrations in triplicate of each analyte. The limits of detection (LOD) and quantification (LOQ) were determined on the basis of signal-to-noise (S/N) ratio of 3 and 10, respectively. LOD and LOQ for each compound were obtained by serial dilutions of stock solution. In order to evaluate the precision, the mixture standard solution was analyzed for six times under the optimal conditions during a single day for intra-day variation, and on three consecutive days for inter-day variation. To assure the repeatability, six different working solutions prepared from the same sample (S1) were assessed. The relative standard deviation (RSD) was chosen to evaluate the repeatability. 

The accuracy of the method was evaluated using a recovery test. The concentration level of the reference standards approximately equivalent to the concentration in the sample were added into a certain amount of the sample (0.10 g), which had been determined previously. The mixture was extracted and then analyzed using the method described previously. The average recoveries were calculated according to the formula: Recovery (%) = (observed amount − original amount)/spiked amount × 100%. The relative standard deviation (RSD) was also chosen to evaluate the recoveries.

### 3.5. Peak Selections and Data Processing

An Xcalibur 2.1 workstation was used for data acquisition and processing. The chemical formulas for all parent and fragment ions of the selected peaks were calculated from the accurate mass using a formula predictor by setting the parameters as follows: C (0–30), H (0–50), O (0–20) and Ring Double Bond (RDB) equivalent value (0–15). Other elements such as N, P, S, Cl and Br were not considered as they are rarely present in this traditional herb. 

## 4. Conclusions

Chemical identification is important during the early stages of drug discovery and development. Here, a sensitive and rapid UHPLC-DAD-ESI-MS/MS method was established, which could be used for simultaneous qualitation and quantitation of chlorogenic acids (CGAs) in Kuding tea. The characteristics of fragmentation pathways and DPIs were first deduced by analyzing six CGA standards in a CID-MS/MS experiment. Then, their respective DPIs could be adopted as the basis for further analysis of the CGAs in the Kuding tea extract. As a result, a total of 68 CGA candidates attributed to 12 categories were unambiguously or preliminarily identified within 18 min chromatographic time. Among them, six CQAs were unambiguously identified by comparison with the reference standards. It was the first systematic report on the distribution of CGAs in Kuding tea. All the results indicated that the established UHPLC-DAD-LTQ-Orbitrap method could be employed as an effective technique to perform and characterize various CGAs from botanic extracts and TCMs.

## Figures and Tables

**Table:** 

**Peak**	**Compound**	**R_1_**	**R_2_**	**R_3_**
13	3-CQA	caffeoyl	H	H
23	5-CQA	H	H	caffeoyl
25	4-CQA	H	caffeoyl	H
41	3,4-diCQA	caffeoyl	caffeoyl	H
42	3,5-diCQA	caffeoyl	H	caffeoyl
44	4,5-diCQA	H	caffeoyl	caffeoyl

note: 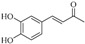 caffeoyl.

**Table 1:** Calibration curves, linearity, LOD and LOQ for 6 investigated CQAs.

Compounds	Regression Equation	Linear Range (μg·mL^−1^)	*r*^2^	LOD (ng)	LOQ (ng)
3-CQA	*Y* = 5349.43*X* − 7565.80	0.978–97.8	1.0000	0.247	0.796
5-CQA	*Y* = 1243.65*X* − 6508.24	1.426–142.6	0.9999	0.132	0.431
4-CQA	*Y* = 2558.74*X* − 17847.34	1.623–162.3	0.9999	0.146	0.597
3,4-DiCQA	*Y* = 1761.60*X* − 13189.53	1.412–141.2	1.0000	1.108	3.232
3,5-DiCQA	*Y* = 1587.92*X* − 10835.65	1.060–106.0	0.9999	0.211	0.674
4,5-DiCQA	*Y* = 1495.94*X* − 15781.81	1.714–171.4	1.0000	0.124	0.479

**Table 2:** Precision, repeatability and recoveries of 6 investigated CQAs.

Compounds	Intra-Day Pecision RSD (%)	Inter-Day Precision	Repeatability RSD (%)	Recovery	
		RSD (%)		Recovery (%)	RSD (%)
3-CQA	1.02	1.45	1.19	101.40	1.99
5-CQA	0.88	1.29	1.23	101.61	2.03
4-CQA	0.79	1.58	1.22	98.62	2.85
3,4-DiCQA	1.74	1.31	1.17	96.40	1.79
3,5-DiCQA	0.91	1.29	1.27	99.88	2.46
4,5-DiCQA	1.08	1.22	1.24	97.95	1.63

**Table 3:** The contents of 6 investigated CQAs in Kuding tea from different origins.

Compounds	Content (mg/g)							
	S1	S2	S3	S4	S5	S6	S7	S8
3-CQA	3.29	4.05	3.53	2.28	4.48	3.37	3.12	4.45
5-CQA	29.74	18.51	18.74	16.32	22.29	30.51	17.93	19.12
4-CQA	2.87	1.99	2.37	2.16	2.69	2.59	4.01	2.89
3,4-DiCQA	5.37	4.26	5.34	5.08	7.84	4.51	1.47	5.04
3,5-DiCQA	37.49	40.17	37.36	45.14	39.02	52.18	23.94	41.98
4,5-DiCQA	28.36	32.15	19.30	27.26	26.19	30.32	29.11	22.72
Total	105.12	98.13	84.64	97.24	99.51	121.48	79.58	93.20

**Table 4:** Characterization of CGAs in Kuding tea using UHPLC-DAD-LTQ-Orbitrap MS.

No.	t_R_/min	Formula [M − H]^−^	Theoretical Mass *m*/*z*	Experimental Mass *m*/*z*	Error/ppm	MS^n^ (*m*/*z*) P-ion (%) ^b^	Identification
1	0.72	C_13_H_21_O_11_	353.1078	353.1080	0.37	MS^2^[353]: 173(100), 191(44), 111(23)	QA-Glc-1
2	0.97	C_13_H_21_O_11_	353.1078	353.1085	1.76	MS^2^[353]: 173(100), 191(41)	QA-Glc-2
3	1.20	C_13_H_21_O_11_	353.1078	353.1090	3.23	MS^2^[353]: 173(100), 191(38), 111(28)	QA-Glc-3
4	2.91	C_22_H_27_O_14_	515.1395	515.1409	2.60	MS^2^[515]: 353(100), 191(49)	CQA-Glc-1
5	2.97	C_28_H_37_O_19_	677.1924	677.1951	4.10	MS^2^[677]: 353(100), 631(54)	CQA-DiGlc-1
6	3.11	C_22_H_27_O_14_	515.1395	515.1415	3.78	MS^2^[515]: 341(100), 179(90), 353(67), 191(27)	CQA-Glc-2
7	3.31	C_28_H_37_O_19_	677.1924	677.1893	4.21	MS^2^[677]: 353(100), 633(30), 515(12.3)	CQA-DiGlc-2
8	3.47	C_22_H_27_O_14_	515.1395	515.1409	2.71	MS^2^[515]: 353(100), 191(81), 179(4)	CQA-Glc-3
9	3.50	C_28_H_37_O_19_	677.1924	677.1934	1.48	MS^2^[677]: 353(100), 335(80), 515(73)	CQA-DiGlc-3
10	3.56	C_22_H_27_O_14_	515.1395	515.1414	3.55	MS^2^[515]: 341(100), 353(89), 179(59), 173(29)	CQA-Glc-4
11	3.60	C_16_H_17_O_9_	353.0867	353.0866	−0.36	MS^2^[353]: 191(100), 179(45), 173(3)	1-CQA
12	3.62	C_28_H_37_O_19_	677.1924	677.1916	−1.04	MS^2^[677]: 353(100), 455(46), 395(35), 515(19)	CQA-DiGlc-4
13 ^Δ^	3.79	C_16_H_17_O_9_	353.0867	353.0869	0.57	MS^2^[353]: 191(100), 179(45), 135(7), 173(4)	3-CQA
14	3.85	C_22_H_27_O_14_	515.1395	515.1410	2.95	MS^2^[515]: 323(100), 191(27), 353(26)	CQA-Glc-5
15	3.96	C_22_H_27_O_14_	515.1395	515.1410	2.95	MS^2^[515]: 353(100), 191(87)	CQA-Glc-6
16	4.03	C_28_H_37_O_19_	677.1924	677.1935	1.75	MS^2^[677]: 353(100), 335(52), 395(37), 515(31)	CQA-DiGlc-5
17	4.11	C_22_H_27_O_14_	515.1395	515.1415	3.78	MS^2^[515]: 353(100), 191(72), 341(69)	CQA-Glc-7
18	4.26	C_22_H_27_O_14_	515.1395	515.1414	3.55	MS^2^[515]: 471(100), 353(18)	CQA-Glc-8
19	4.29	C_32_H_35_O_17_	691.1869	691.1884	2.23	MS^2^[691]: 353(100), 673(12)	CFQA-Glc-1
20	4.35	C_16_H_17_O_8_	337.0928	337.0920	0.55	MS^2^[337]: 163(100), 191(7), 173(5)	3-*p*CoQA
21	4.39	C_22_H_27_O_14_	515.1395	515.1410	2.95	MS^2^[515]: 353(100), 529(12)	CQA-Glc-9
22	4.39	C_32_H_35_O_17_	691.1869	691.1880	1.70	MS^2^[691]: 673(100), 529(86), 367(15)	CFQA-Glc-2
23 ^Δ^	4.52	C_16_H_17_O_9_	353.0867	353.0868	0.15	MS^2^[353]: 191(100), 179(3)	5-CQA
24	4.58	C_32_H_35_O_17_	691.1869	691.1882	−1.87	MS^2^[691]: 529(100), 353(11), 367(5)	CFQA-Glc-3
25 ^Δ^	4.68	C_16_H_17_O_9_	353.0867	353.0868	0.23	MS^2^[353]: 173(100), 179(57), 191(27), 135(8)	4-CQA
26	4.75	C_17_H_19_O_9_	367.1024	367.1024	0.00	MS^2^[367]: 193(100), 173(4)	3-FQA
27	5.31	C_16_H_17_O_8_	337.0928	337.0916	−0.72	MS^2^[337]: 191(100), 163(4), 173(1)	5-*p*CoQA
28	5.71	C_17_H_19_O_9_	367.1024	367.1035	3.16	MS^2^[367]: 191(100), 173(5), 191(2)	5-FQA
29	5.81	C_31_H_33_O_17_	677.1712	677.1722	1.50	MS^2^[677]: 353(100), 515(13)	DiCQA-Glc-1
30	5.91	C_16_H_17_O_8_	337.0928	337.0915	−0.99	MS^2^[337]: 173(100), 191(75), 163(8)	4-*p*CoQA
31	5.91	C_31_H_33_O_17_	677.1712	677.1722	1.50	MS^2^[677]: 353(100), 515(85)	DiCQA-Glc-2
32	6.10	C_31_H_33_O_17_	677.1712	677.1720	1.14	MS^2^[677]: 515(100), 353(65)	DiCQA-Glc-3
33	6.20	C_17_H_19_O_9_	367.1024	367.1034	2.73	MS^2^[367]: 173(100), 134(9), 193(3)	4-FQA
34	6.28	C_31_H_33_O_17_	677.1712	677.1724	1.76	MS^2^[677]: 515(100), 353(20)	DiCQA-Glc-4
35	6.48	C_31_H_33_O_17_	677.1712	677.1727	2.22	MS^2^[677]: 515(100), 353(40)	DiCQA-Glc-5
36	6.58	C_31_H_33_O_17_	677.1712	677.1713	0.05	MS^2^[677]: 515(100), 353(46)	DiCQA-Glc-6
37	6.72	C_31_H_33_O_17_	677.1712	677.1692	−2.92	MS^2^[677]: 515(100), 609(10), 353(9)	DiCQA-Glc-7
38	7.23	C_31_H_33_O_17_	677.1712	677.1724	1.67	MS^2^[677]: 609(100), 515(89), 353(24)	DiCQA-Glc-8
39	7.46	C_31_H_33_O_17_	677.1712	677.1725	1.95	MS^2^[677]: 515(100), 631(36)	DiCQA-Glc-9
40	7.53	C_25_H_23_O_12_	515.1184	515.1200	3.10	MS^2^[515]: 353(100), 191(34) MS^3^[515]: 191(100), 179(76), 111(41)	1,3-DiCQA
41 ^Δ^	7.99	C_25_H_23_O_12_	515.1184	515.1188	0.71	MS^2^[515]: 353(100), 173(24) MS^3^[353]: 173(100), 179(68), 191(46)	3,4-DiCQA
42 ^Δ^	8.57	C_25_H_23_O_12_	515.1184	515.1201	3.33	MS^2^[515]: 353(100), 191(1) MS^3^[353]: 191(100), 179(48), 135(12)	3,5-DiCQA
43	9.61	C_25_H_23_O_12_	515.1184	515.1200	3.10	MS^2^[515]: 353(100), 191(38) MS^3^[353]: 191(100), 179(73), 111(31)	*Cis*-1,3-DiCQA
44 ^Δ^	9.96	C_25_H_23_O_12_	515.1184	515.1187	0.60	MS^2^[515]: 353(100), 191(8) MS^3^[353]: 173(100), 191(89), 179(81)	4,5-DiCQA
45	10.39	C_25_H_23_O_11_	499.1235	499.1255	3.95	MS^2^[499]: 353(100), 335(25), 173(10), 179(8)	*p*CoCQA-1
46	10.91	C_25_H_23_O_11_	499.1235	499.1255	4.07	MS^2^[499]: 337(100), 173(32), 335(14), 353(4)	*p*CoCQA-2
47	10.91	C_34_H_29_O_15_	677.1501	677.1518	2.50	MS^2^[677]: 515(100), 497(30)	TriCQA-1
48	11.17	C_25_H_23_O_12_	515.1184	515.1176	−1.54	MS^2^[515]: 353(100) MS^3^[353]: 191(100), 179(19), 135(9)	1,5-DiCQA
49	11.18	C_26_H_25_O_12_	529.1341	529.1357	3.08	MS^2^[529]: 353(100), 367(69)	CFQA-1
50	11.24	C_25_H_23_O_11_	499.1235	499.1254	3.77	MS^2^[499]: 337(100), 335(8), 163(7)	*p*CoCQA-3
51	11.60	C_25_H_23_O_11_	499.1235	499.1255	4.07	MS^2^[499]: 353(100), 337(13), 191(4), 179(2)	*p*CoCQA-4
52	11.82	C_26_H_25_O_12_	529.1341	529.1354	2.51	MS^2^[529]: 367(100), 173(28), 179(5)	CFQA-2
53	12.16	C_25_H_23_O_11_	499.1235	499.1252	3.45	MS^2^[499]: 353(100), 337(48), 335(3)	*p*CoCQA-5
54	12.65	C_26_H_25_O_12_	529.1341	529.1354	2.62	MS^2^[529]: 367(100), 193(5)	CFQA-3
55	12.71	C_34_H_29_O_15_	677.1501	677.1513	1.79	MS^2^[677]: 515(100), 497(40)	TriCQA-2
56	13.11	C_26_H_25_O_12_	529.1341	529.1352	2.26	MS^2^[529]: 353(100), 367(36), 191(9), 179(6)	CFQA-4
57	13.16	C_34_H_29_O_15_	677.1501	677.1512	1.60	MS^2^[677]: 515(100), 497(35), 659(21)	TriCQA-3
58	13.57	C_26_H_25_O_12_	529.1341	529.1349	1.70	MS^2^[529]: 367(100)	CFQA-5
59	13.84	C_25_H_23_O_11_	499.1235	499.1252	3.45	MS^2^[499]: 337(100), 173(10), 335(4), 179(2)	*p*CoCQA-6
60	14.25	C_34_H_29_O_15_	677.1501	677.1514	1.88	MS^2^[677]: 515(100), 497(29)	TriCQA-4
61	14.35	C_25_H_23_O_11_	499.1235	499.1251	3.27	MS^2^[499]: 353(100), 337(8)	*p*CoCQA-7
62	14.45	C_34_H_29_O_15_	677.1501	677.1506	0.70	MS^2^[677]: 515(100), 617(66)	TriCQA-5
63	14.73	C_26_H_25_O_12_	529.1341	529.1352	2.15	MS^2^[529]: 353(100), 367(20)	CFQA-6
64	14.80	C_25_H_23_O_12_	515.1184	515.1199	2.85	MS^2^[515]: 353(100), 173(3), 179(2) MS^3^[353]: 173(100), 179(84), 191(81), 135(13)	1,4-DiCQA
65	15.21	C_26_H_25_O_12_	529.1341	529.1355	2.73	MS^2^[529]: 353(100), 367(21), 203(10), 335(6)	CFQA-7
66	16.38	C_26_H_25_O_12_	529.1341	529.1354	2.51	MS^2^[529]: 367(100), 179(17)	CFQA-8
67	16.96	C_26_H_25_O_12_	529.1341	529.1352	2.15	MS^2^[529]: 367(100), 353(44)	CFQA-9
68	16.96	C_34_H_29_O_15_	677.1501	677.1508	0.98	MS^2^[677]: 515(100), 353(7)	TriCQA-6

^Δ^ Identified by comparison with reference standards.

## References

[1] Jiangsu New Medical College (1986). Dictionary of Traditional Chinese Medicine.

[2] Liu L.X., Sun Y., Laura T., Liang X.F., Ye H., Zeng X.X. (2009). Determination of polyphemolic content and antioxidant activity of kudingcha made from *Ilex Kudingcha* C.J. Tseng. Food Chem..

[3] Thuong P.T., Su N.D., Ngoc T.M., Hung T.M., Dang N.H., Thuan N.D., Bae K.H., Oh W.K. (2009). Antioxidant activity and principles of Vietnam bitter tea *Ilex Kudingcha*. Food Chem..

[4] Fan S.G., Zhang Y., Hu N., Sun Q.H., Ding X.B., Li G.W., Zheng B., Gu M., Huang F.S., Sun Y.Q. (2012). Extract of Kuding tea prevents high-fat diet-induced metabolic disorders in C57BL/6 mice via liver X receptor (LXR) β antagonism. PLoS ONE.

[5] Song C.W., Xie C., Zhou Z.W., Yu S.G., Yu S.G., Fang N.B. (2012). Antidiabetic effect of an active components group from *Ilex Kudingcha* and its chemical composition. Evid. Based Complement. Altern. Med..

[6] Xu D., Wang Q., Zhang W., Hu B., Zhou L., Zeng X., Sun Y. (2015). Inhibitory activities of caffeoylquinic acid derivatives from *Ilex kudingcha* C.J. Tseng on α-glucosidase from Saccharomyces cerevisiae. J. Agric. Food Chem..

[7] Song J.L., Qian Y., Li G.J., Zhao X. (2013). Anti-inflammatory effects of kudingcha methanol extract (*Ilex Kudingcha* C.J. Tseng) in dextran sulfate sodium-induced ulcerative colitis. Mol. Med. Rep..

[8] Zheng J., Zhou H.Y., Zhao Y.F., Lun Q.X., Liu B.L., Tu P.F. (2015). Triterpenoid-enriched extract of *Ilex Kudingcha* inhibits aggregated LDL-induced lipid deposition in macrophages by downregulating low density lipoprotein receptor-related protein 1 (LRP1). J. Funct. Foods.

[9] Kai Z., Li G.J., Sun P., Wang R., Qian Y., Zhao X. (2014). In vitro and in vivo anti-cancer activities of Kuding tea (*Ilex Kudingcha* C.J. Tseng) against oral cancer. Exp. Ther. Med..

[10] Clifford M.N., Marks S., Knight S., Kuhnert N. (2006). Characterization by LC-MS(n) of four new classes of *p*-coumaric acid-containing diacyl chlorogenic acids in green coffee beans. J. Agric. Food Chem..

[11] Michael N.C. (2000). Chlorogenic acids and other cinnamates-nature, occurrence, dietary burden, absorption and metabolism. J. Sci. Food Agric..

[12] Wang Q.C., Zhang X., Zhang W.Q., Sun X.Q., Hu B., Sun Y., Zeng X.X. (2013). Purification and HPLC analysis of caffeoylquinic acids from Kudingcha made from *Ilex Kudingcha* C.J. Tseng. Food Sci..

[13] Liu A.Y., Che Y.Y., Wang F., Shang Z.P., Lu J.Q., Dai S.Y., Zhang J.Y., Cai W. (2016). Identification of metabolites of 6′-hydroxy-3,4,5,2′,4′-pentamethoxychalcone in rats by a combination of ultra-high-performance liquid chromatography with linear ion trap-Orbitrap mass spectrometry based on multiple data processing techniques. Molecules.

[14] Sun M.H., Luo Z.Q., Liu Y., Yang R.R., Lu L., Yu G.H., Ma X.Y., Liu A.X., Guo Y.F., Zhao Y.H. (2016). Identification of the major components of *Buddleja officinalis* extract and their metabolites in rat urine by UHPLC-LTQ-Orbitrap. J. Food Sci..

[15] Zhang J.Y., Wang Z.J., Li Y., Liu Y., Cai W., Li C., Lu J.Q., Qiao Y.J. (2016). A strategy for comprehensive identification of sequential constituents using ultra-high-performance liquid chromatography coupled with linear ion trap-Orbitrap mass spectrometer, application study on chlorogenic acids in Flos Lonicerae Japonicae. Talanta.

[16] Wang T.H., Zhang J., Qiu X.H., Bai J.Q., Gao Y.H., Xu W. (2016). Application of Ultra-High-Performance liquid chromatography Coupled with LTQ-Orbitrap Mass spectrometry for the qualitative and quantitative analysis of *Polygonum multiflorum* Thumb. and its processed products. Molecules.

[17] Zhang J.X., Guan S.H., Sun J.H., Liu T., Chen P., Feng R.H., Chen X., Wu W.Y., Yang M., Guo D.A. (2015). Characterization and profiling of phenolic amides from Cortex Lycii by ultra-high performance liquid chromatography coupled with LTQ-Orbitrap mass spectrometry. Anal. Bioanal. Chem..

[18] Makarov A., Denisov E., Lange O., Horning S. (2006). Dynamic range of mass accuracy in LTQ Orbitrap hybrid mass spectrometer. J. Am. Soc. Mass Spectrom..

[19] Zhang J.Y., Zhang Q., Li N., Wang Z.J., Lu J.Q., Qiao Y.J. (2013). Diagnostic fragment-ion-based and extension strategy coupled to DFIs intensity analysis for identification of chlorogenic acids isomers in Flos Lonicerae Japonicae by HPLC-ESI-MS^n^. Talanta.

[20] Schram K., Miketova P., Slanina J., Humpa O., Taborska E. (2004). Mass spectrometry of 1,3- and 1,5-dicaffeoylquinic acids. J. Mass Spectrom..

[21] Clifford M.N., Johnson K.L., Knight S., Kuhnert N. (2003). Hierarchical scheme for LC-MS^n^ identification of chlorogenic acids. J. Agric. Food Chem..

[22] Wang Z., Clifford M.N. (2008). Comparison of the profiles chlorogenic acids and their derivatives from three Chinese traditional herbs by LC-MS^n^. Acta Pharm. Sin..

[23] Wang J., Wang N.L., Yao X.S., Susumu K.A. (2006). Caffeoylquinic acid derivatives from *Bidens parviflora* and their antihistamine release activities. Chin. Tradit. Herb. Drugs.

